# The Impact of Organic Selenium (IV) on *Hypericum perforatum* L. under Cadmium Stress and Non-Stress Conditions

**DOI:** 10.3390/plants13152099

**Published:** 2024-07-29

**Authors:** Joanna Giebułtowicz, Joanna Ślusarczyk, Sylwia Wyderska, Piotr Wroczyński

**Affiliations:** 1 Department of Drug Chemistry, Pharmaceutical and Biomedical Analysis, Medical University of Warsaw, Banacha 1, 02-097 Warsaw, Poland; s.wyderska@gmail.com (S.W.); piotr.wroczynski@wum.edu.pl (P.W.); 2 Department of Environmental Biology, Institute of Biology, Jan Kochanowski University, Uniwersytecka 7, 25-406 Kielce, Poland; joanna.slusarczyk@ujk.edu.pl

**Keywords:** selenium, cadmium, environment, Selol, St. John’s wort

## Abstract

The issue of soil contamination by heavy metals is widely acknowledged. Some plants, including medicinal species like St. John’s wort (*Hypericum perforatum* L.), exhibit accumulation traits, allowing them to accumulate elevated levels of metals, e.g., cadmium (Cd), within their cells. Selenium (Se) may increase the tolerance of plants to abiotic stress caused by the presence of heavy metal in the environment. Depending on its form (oxidation state, organic/inorganic), Se influences plant growth, secondary metabolite content, and biotic stress, as well as incorporates into shoots, providing economic and health benefits for consumers. So far, there are no data on the influence of organic Se(IV) on plants. Our study aimed to determine the effect of organic Se(IV) on the growth, active compound levels (anthranoids, polyphenols), and ultrastructure of St. John’s wort without and under cadmium stress. The phytochemical analysis and microscopic examination was performed on shoots from different days of St. John’s wort in vitro culture on a few variants of Murashige and Skoog medium with Cd (25 and 400 µM) and/or organic Se (IV). Exposure to Se(IV) did not affect hypericins but increased the polyphenol content in the shoots and the biomass. Se(IV) caused an increase in starch grain number in chloroplasts, whereas Cd exposure resulted in the degradation of the chloroplast structure, increased cell vacuolation, as well as swollen mitochondrial cristae. The addition of Se(IV) to these combinations reduced the degree of degradation and growth inhibition and a high content of Se(IV) in plants was observed. Se(IV) had no impact on Cd content at environmental Cd concentrations, but showed an effect at extremely high Cd concentrations. Thus, organic Se(IV) has a beneficial effect on St. John’s wort growth, polyphenol content, and incorporation in shoots and prevents Cd toxicity. Media enriched with organic Se(IV) have both economic advantages and health benefits due to a higher plant growth rate and increased concentrations of polyphenols with strong antioxidant properties, relatively enriched with Se. However, organic Se(IV) should be used with caution in polluted areas. In perspective, speciation analysis and molecular study are crucial to understand the fate and effect of Se (IV) on plants.

## 1. Introduction

Phytotherapy is one of the most popular types of alternative medicine because it is widely available (sold without prescription) and not expensive. St. John’s wort (*Hypericum perforatum* L.) has a rich history of use spanning centuries for treating various medical conditions [1]. Aqueous extracts of *Hyperici herba* find application in liver and gall bladder diseases linked to inadequate bile secretion, as well as in spastic conditions within the gastrointestinal tract [2]. Alcoholic extracts are commonly used for mild to moderate depression [2]. The broad therapeutic efficacy of St. John’s wort is attributed to the fact that St John’s wort biosynthesizes several types of biologically active compounds like flavonoid-quercetin derivatives—hyperoside, isoquercitrin, rutoside, essential oil (pinen) and dianthrone derivates—hypericin and pseudohypericin, catechin tannins, and leucocyanidines [2].

Herbal medicines are wrongly considered to be always safe and have no side effects. However, besides all other side effects caused by the herbs’ active compounds, those related to high heavy metal content should be considered as well. Some plants, including the medicinal plant *Hypericum perforatum* L., are accumulators, capable of amassing relatively high concentrations of metals, e.g., cadmium (Cd), in their cells [3] even ranging from 1 to 5% of their biomass [4]. The presence of cadmium in the environment poses a significant threat to human health, primarily due to its potential to cause adverse health effects. Elevated Cd exposure has been linked to renal tubular dysfunction, osteomalacia, osteoporosis, as well as disorders in glucose metabolism, and increased risks of breast and lung cancer, cerebral infarction, and cardiac failure. Therefore, new methods are being sought to reduce the concentration of Cd in plants and soil [5,6].

Cd is widely recognized as one of the most toxic and mobile elements in the ecological landscape. Sources of Cd in the environment are diverse, including natural geological processes and human activities. Soil contamination is typically indicated by Cd concentrations exceeding 3 mg/kg, although some regions may naturally have higher levels independent of human influence [7]. Cd also interferes with various physiological and biochemical processes in plants. On a molecular level, it can disrupt enzyme activities, impair photosynthesis, hinder nutrient uptake, disturb water balance, and cause oxidative stress. Cd induces osmotic stress and reduces water content, stomatal conductance, and transpiration in plants causing leaf rolling. Furthermore, it reduces the uptake of essential nutrients like iron and zinc, leading to leaf chlorosis. Cd inhibits plant growth, affects carbon fixation, and chlorophyll content, and can cause necrosis [8].

*Hypericum perforatum* L. can also accumulate in selenium (Se), whose intake depends on its concentration in soil. In Europe, selenium-poor soils are prevalent, especially in Germany, Denmark, Scotland, Finland, and certain Balkan countries. Climate change further contributes to declining Se levels. Consequently, some countries utilize Se-enriched fertilizers [9] to enhance crop yields. Se, an essential trace element for humans and animals, has not yet been confirmed as an essential nutrient for plants. Nevertheless, Se has been shown to stimulate plant growth [10], delay the ageing process, and protect against abiotic stresses such as low temperature [11], drought [12], salinity, [13] and exposure to heavy metals [14]. It can accumulate in shoots, providing economic and health benefits for consumers [15]. Moreover, Se has been shown to ameliorate the toxic effects of Cd, with the degree of stress tolerance influenced by its form [16]. Organic Se (II) (selenomethionine, SeMet) exhibits distinct distribution and physiological effects compared to Se inorganic forms, being more readily absorbed by plants. SeMet was more effective in reducing Cd concentration and enhancing Se concentration in the roots and shoots of rice seedlings, as well as in regulating antioxidant enzyme activities, compared to inorganic selenium [15]. Similar observation was made on garlic, where organic Se treatment caused 1.8–3.9 times higher Se and 1.1–8.8 times reduced Cd content in garlic bulbs comparing to inorganic Se treatments [17]. Other reports consistently revealed that the uptake efficiency of SeMet was much higher than that of inorganic forms [18,19,20]. Notably, organic Se (IV, Selol), unlike inorganic Se, enhances the biomass production of Lion’s Mane and protects the mycelium under inorganic Se stress [21]. However, there are currently no available data on the impact of organic Se (IV, Selol) specifically on mitigating the toxic effects of Cd.

Selol, a mixture of seleno-triglycerides derived from the chemical modification of sunflower oil, represents the sole organic source of Se (IV). While the impact of inorganic and organic Se (II), such as Se-methylselenocysteine, on plants has been studied across various species, [15,16,22,23,24], there is a notable gap in understanding the influence of organic selenium (IV, Selol) on plant growth, ultrastructure, and secondary metabolite levels.

To fill this gap, the study aimed to assess in in vitro study the impact of organic Se (IV) on growth, ultrastructure, and the levels of secondary metabolites (anthranoids, polyphenols) of *Hypericum perforatum* L. (a) cultured in control media, (b) cultured in media with environmental concentrations of Cd (25 µM), and (c) cultured in media with extremely high Cd levels (400 µM). We also assessed the effect of Se (IV) on cadmium intake.

## 2. Results

### 2.1. Phytochemical Analysis

**Figure 1:**
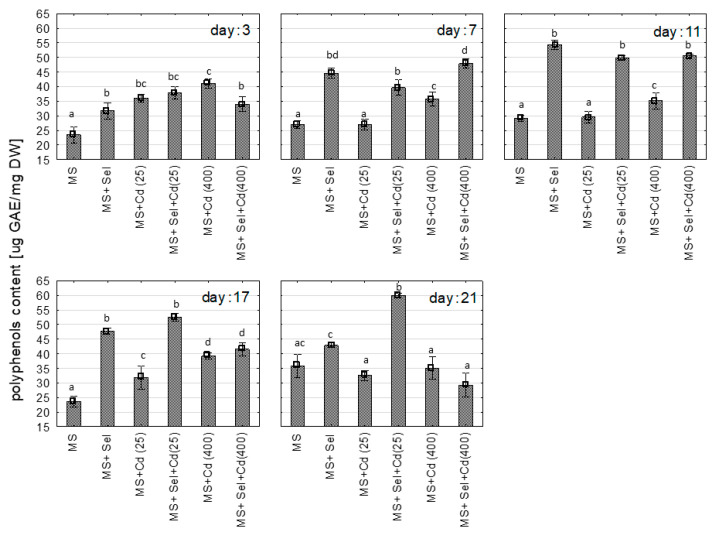
The polyphenolic content [µg gallic acid equivalents (GAE)/mg dry weight] in methanolic extracts (50%aq) of St John*’*s wort cultured in vitro for 3, 7, 11, 17, and 21 days on various medium modifications. Groups identified by the same letter on the same cultural day do not demonstrate statistically significant differences using ANOVA with *p* = 0.05. Abbreviations: MS + Cd(25)*—*MS medium with 25 µM Cd; MS + Cd (400)*—*MS medium with 400 µM Cd; Sel*—*Selol, Se (IV).

The exposure to Cd or Se did not affect the concentration of hypericins (*p* > 0.05). At the 17th day of culture, the hypericins level was 0.19 ± 0.04 mg/g (MS); 0.36 ± 0.30 mg/g (MS with 25 µM of Cd), 0.28 ± 0.14 mg/g (MS with 400 µM of Cd); 0.22 ± 0.06 mg/g (MS with 25 µM of Cd and Se), and 0.17 ± 0.04 mg/g (MS with 400 µM of Cd with Se). The exposure to Cd or Se increased the polyphenol content in the shoots (Figure 1). For Cd, the increase was in a dose-dependent manner. When the plant was exposed to Se and Cd simultaneously, the highest differences between media with and without Se was observed for lower cadmium level (25 µM). The interactions between Cd and Se were confirmed using a two-way analysis of variance (*p* = 0.00651 for the 3rd day, *p* = 0.00003 for the 17th day, *p* = 0.00005 for the 21st day).

**Figure 2:**
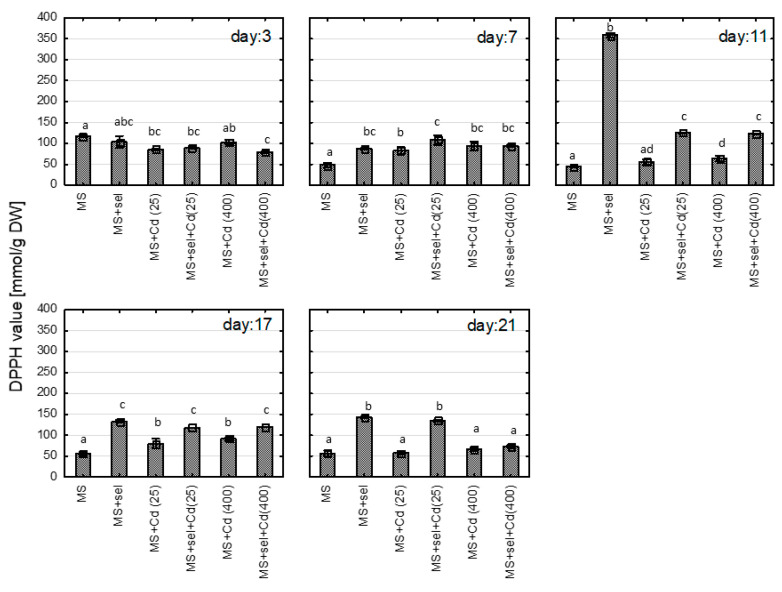
The DPPH value [mM Trolox equivalents/g dry weight] in methanolic extracts (50%aq) of St John’s wort cultured in vitro for 3, 7, 11, 17, and 21 days on various medium modifications. Groups identified by the same letter on the same cultural day do not demonstrate statistically significant differences using ANOVA with *p* = 0.05. Abbreviations: MS + Cd(25)—MS medium with 25 µM Cd; MS + Cd (400)—MS medium with 400 µM Cd; Sel—Selol*,* Se (IV).

The changes in polyphenol concentration were similar to those in DPPH values (Figure 2). The addition of Se (IV, Selol) to the culture medium also increased the DPPH value compared to the control (MS). Moreover, the Se enrichment medium with Cd resulted in an increase in DPPH value in the shoots’ starting from 7th day of culture compared to the medium with the Cd only (MS + Cd). However, the interaction was only confirmed by ANOVA for the 21st day of culture (*p* < 0.00001).

The Se concentration in shoots, after the 21st day of culture, was 550 ± 70 μg/g, which confirmed the easy absorption of Se from its organic form by *Hypericum perforatum* L. plants. The content of Cd was 46 ± 38 μg/g in plants cultured in media with 25 µM of Cd. Organic Se (IV) did not affect the level of Cd, which was equal to 46 ± 14 μg/g. Different results, i.e., a significant influence of Se (IV) on Cd level in shoots, were observed for 400 µM of the Cd level, where the concentration of Cd was more than 2.5 times higher (1010 ± 190 μg/g vs. 2600 ± 61 μg/g, *p* < 0.00001) in cultures with Se.

### 2.2. Growth Rates

**Figure 3:**
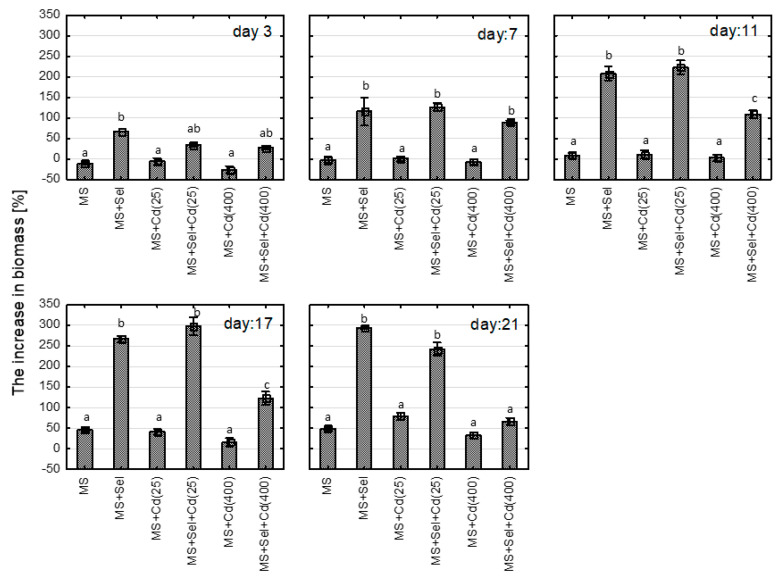
The increase in biomass [%] of St John*’*s wort cultured in vitro for 3, 7, 11, 17, and 21 days on various medium modifications. Groups identified by the same letter on the same cultural day do not demonstrate statistically significant differences using ANOVA with *p* = 0.05. Abbreviations: MS + Cd(25)*—*MS medium with 25 µM Cd; MS + Cd (400)*—*MS medium with 400 µM Cd; Sel*—*Selol, Se (IV).

**Figure 4:**
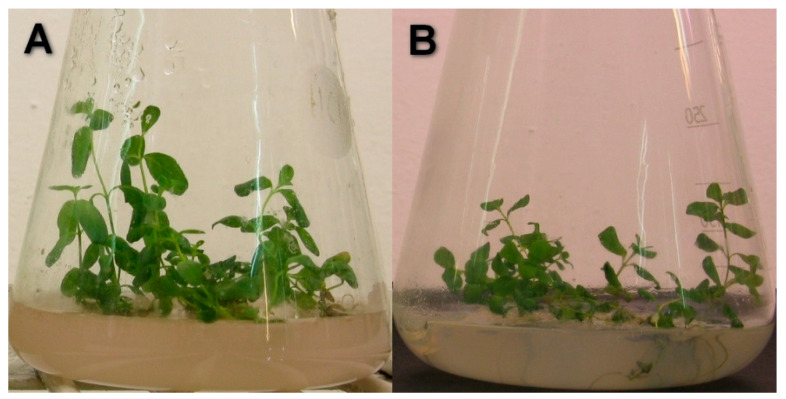
Effect of Se (IV) on the grow of St John*’*s wort. *Hypericum perforatum* L. cultured in various variants of MS medium on 21st day of culture. (**A**) Plant cultured in MS medium with Se, (**B**) plant cultured in MS medium.

**Figure 5:**
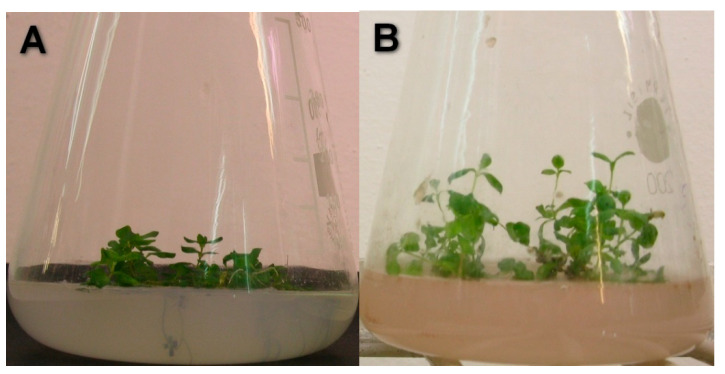
Effect of Se (IV) on the grow of St John*’*s wort exposed to environmental Cd concentration. *Hypericum perforatum* L. cultured in MS medium with Cd (25 µM) on 17th day of culture. (**A**) Plant cultured in MS medium with Cd (25 µM), (**B**) plant cultured in MS medium with Cd (25 µM) with Se (IV).

The relative growth rates were not influenced by Cd (Figure 3). Simultaneously, organic Se (IV) caused significantly higher growth rates starting from the 3rd day of the culture (*p* < 0.00001) (Figure 4. Moreover, the addition of Se to the medium with Cd resulted in enhanced relative growth rates starting from the 7th day of culture. The interactions between Cd and Se were confirmed using two-way analysis of variance (*p* = 0.01010 for 7th day, *p* = 0.00019 for 11th day, *p* < 0.00001 for 17th and 21st day). The plants grown in a medium with Se have a higher leaf surface and thicker shoots (Figure 5).

### 2.3. Microscopic Examination

#### 2.3.1. Polyphenols Content

Microscope observations confirmed the previously described differences in polyphenol content. The increase in the number of polyphenols in the leaf cells of plants cultured in the media with Se compared to the respective media without Se was observed both after 7 and 17 days of culture. The addition of 1% caffeine [25] in the procedure of preparation of the resin-embedded samples was performed to stabilize these compounds in the cells.

**Figure 6:**
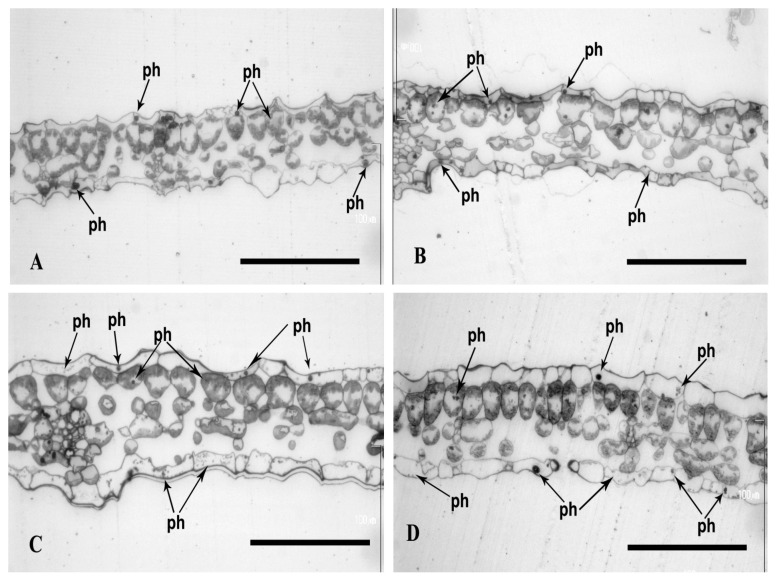
Light micrographs of leaf cross-sections of plants with St. John*’*s wort after 7 and 17 days of culture. Semi-thin sections were stained in toluidine blue. Bar = 100 μm; (**A**) MS medium after 7 days of culture, (**B**) MS medium after 17 days of the culture, (**C**) MS medium + Se after 7 days of culture, (**D**) MS medium + Se after 17 days of culture; ph*—*polyphenols.

**Figure 7:**
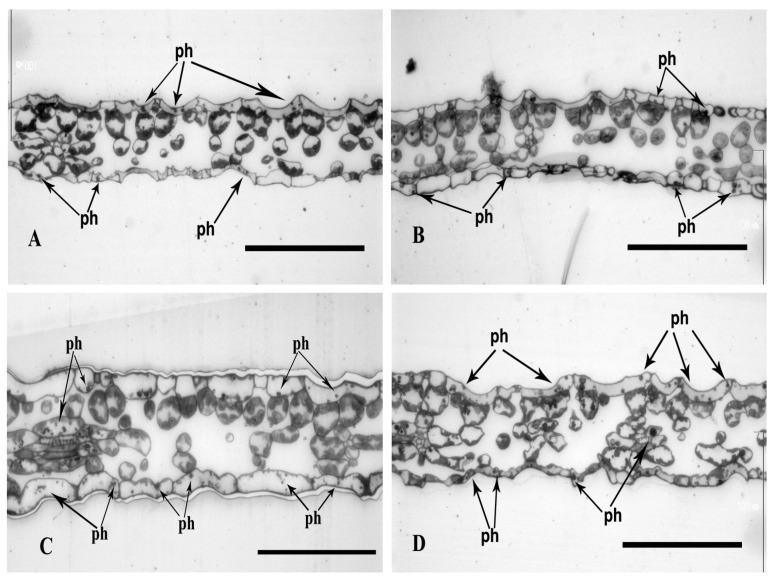
Light micrographs of leaf cross-sections of plants with St. John*’*s wort after 7 and 17 days of culture in MS medium with Cd (25 µM). Semi-thin sections were stained in toluidine blue. Bar = 100 μm; (**A**) MS medium + 25 µM Cd after 7 days of culture, (**B**) MS medium + 25 µM Cd after 17 days of culture, (**C**) MS medium + Se + 25 µM Cd after 7 days of culture, (**D**) MS medium + Se + 25 μM Cd after 17 days of culture; ph*—*polyphenols.

The polyphenols in the semi-thin cross-sections of the leaves of the plants cultured in a control media were observed as black spherical deposits in the upper and lower epidermis. They also occurred in a smaller amount in the palisade parenchyma cells (Figure 6A,C). The leaves of plants cultured in a medium variation, MS + Se, revealed a higher polyphenol concentration in the epidermis compared to the control (MS). The polyphenols were also present in the palisade and spongy parenchyma (Figure 6A–D). The leaves of plants cultured in media with Cd also revealed higher polyphenol content (comparing to control) visible as black spherical deposits and small granules in the upper and lower epidermis. More frequently, they were observed in the lower epidermis than the upper epidermis. Occasionally, they were also present in the cells of the parenchyma (Figure 7A–D).

**Figure 8:**
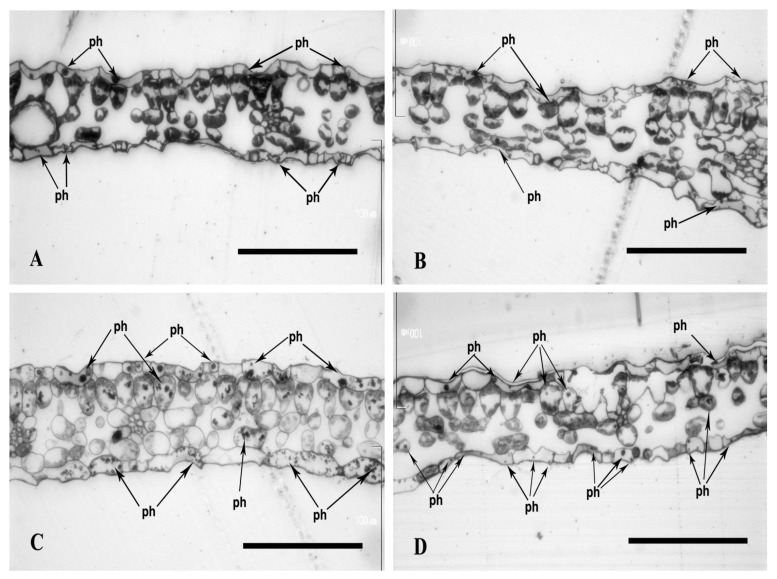
Light micrographs of leaf cross-sections of plants with St. John*’*s wort after 7 and 17 days of culture in MS medium with Cd (400 µM). Semi-thin sections were stained in toluidine blue. Bar = 100 μm; (**A**) MS medium + 400 μM Cd after 7 days of culture. (**B**) MS medium + 400 μM Cd after 17 days of culture. (**C**) MS medium + Se + 400 μM Cd after 7 days of culture. (**D**) MS medium + Se + 400 μM Cd after 17 days of culture; ph*—*polyphenols.

The media with both Se and Cd (25 µM), but not with Cd (400 µM), increased polyphenol content compared to those with only Se or Cd. In all cases of media with Cd and Se, very large numbers of black beads of various sizes in the cells of all tissues of leaves were observed (Figure 8A,B in comparison to Figure 8C,D).

#### 2.3.2. Increase in Biomass

Microscope observations confirmed the previously described higher increase in biomass of plants cultured in media with Se than without Se. The difference between the combinations was also noted after 17 days of growth.

The leaf blade thickness of plants cultured in media (MS) with Se (Figure 6C,D) was up to 50% higher compared to the control media (78 ± 12 microns after 7 days and 83 ± 10 microns after 17 days in MS medium + Se; 53 ± 10 microns after 7 days of culture and 65 ± 12 microns after 17 days (Figure 6A,B), *p* < 0.00001, *p* = 0.00006). A similar corelation was observed for the media with Cd (25 µM). The leaf blade thickness of plants cultured in media with Se was about 30% greater than in those without Se (74 ± 6 microns after 7 days and 79 ± 11 microns after 17 days in MS medium + 25 µM Cd + Se; and 58 ± 6 microns after 7 days, and 61 ± 11 microns after 17 days in MS medium + 25 µM Cd (Figure 7A–D), *p* = 0.00031; *p* = 0.00008). However, the Se addition to the media with Cd (400 µM) revealed significantly higher blade thickness only after 7 days of culture (approx. 10%). The thickness after 7 and 17 day of culture was 71 ± 4 microns and 72 ± 11 microns in media with Se and Cd (400 µM) (Figure 8C,D) and 63 ± 7 microns and 74 ± 16 microns in media only with Cd (*p* = 0.04987, *p* = 0.72) (Figure 8A,B). The interaction between Se and Cd was confirmed by ANOVA for 7 and 17 days of culture (*p* = 0.04997, *p* = 0.1919).

#### 2.3.3. Ultrastructural Differences

**Figure 9:**
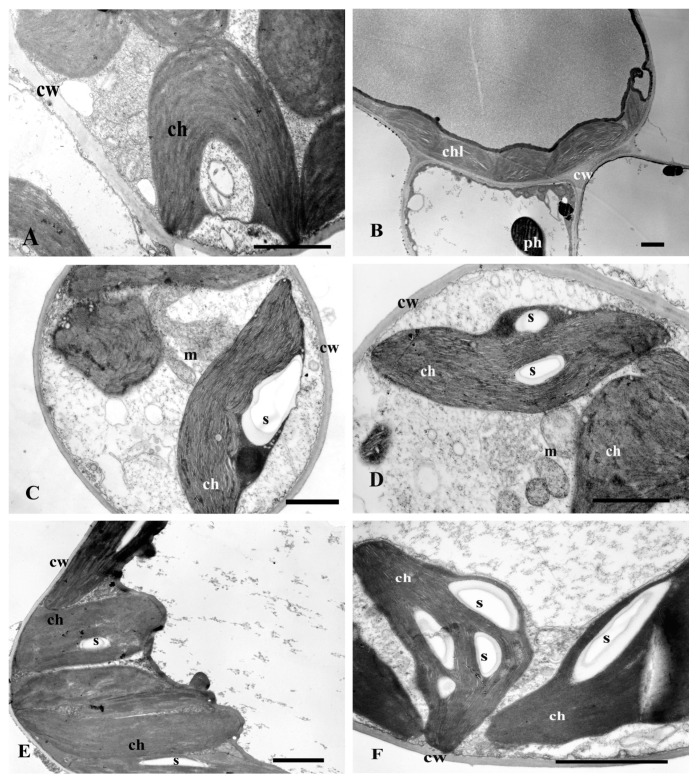
Electron micrographs of leaf spongy parenchyma cells of St. John’s wort after 7 and 17 days of culture on various media modifications (MS or MS + Se). Description in the text. (**A**,**B**)—MS medium after 7 days of culture. (**C**,**D**)—MS medium with Se after 7 days of culture. (**E**)—MS medium after 17 days of culture. (**F**)—MS medium + Se after 17 days of culture. Bar = 1 μm; ch, chl—chloroplast, cw—cell wall, m—mitochondrion, ph—phenolic deposits, s—starch grain.

The ultra-thin sections revealed some differences in ultrastructure of leaves from the plants cultured in the various combinations, which correspond to the changes described above in the growth of the biomass and polyphenol level. The leaf cells of plants cultured in MS medium for 7 days had a typical leaf parenchyma ultrastructure with a plurality of chloroplast located along the plant cell’s wall. The chloroplasts had poorly separated thylakoids and sometimes a characteristic cup shape of C (Figure 9A). Rarely, the starch grains in them were observed. The numerous mitochondria with a typical structure (Figure 9A) were located near the chloroplasts. Sometimes in vacuoles of parenchyma and of epidermis cells, the polyphenols as spherical black osmiophilic deposits were observed. (Figure 9B).

After 17 days of culture, the cells were more vacuolated. Lenticular-shaped chloroplasts were located along the plant cell’s wall. Their thylakoids were poorly separated, and between them some starch grains were present (Figure 9E). In the cytoplasm, single spherical deposits of phenols were observed.

The leaf cells of plants cultured in MS + Se medium revealed more phenolic compounds in the cytoplasm and more starch grains in typical structured chloroplasts both after 7 (Figure 9C,D) and 17 days of culture (Figure 9F) compared to the control (MS). In this media variation, more typical structured mitochondria were observed as well (Figure 9C,D).

**Figure 10:**
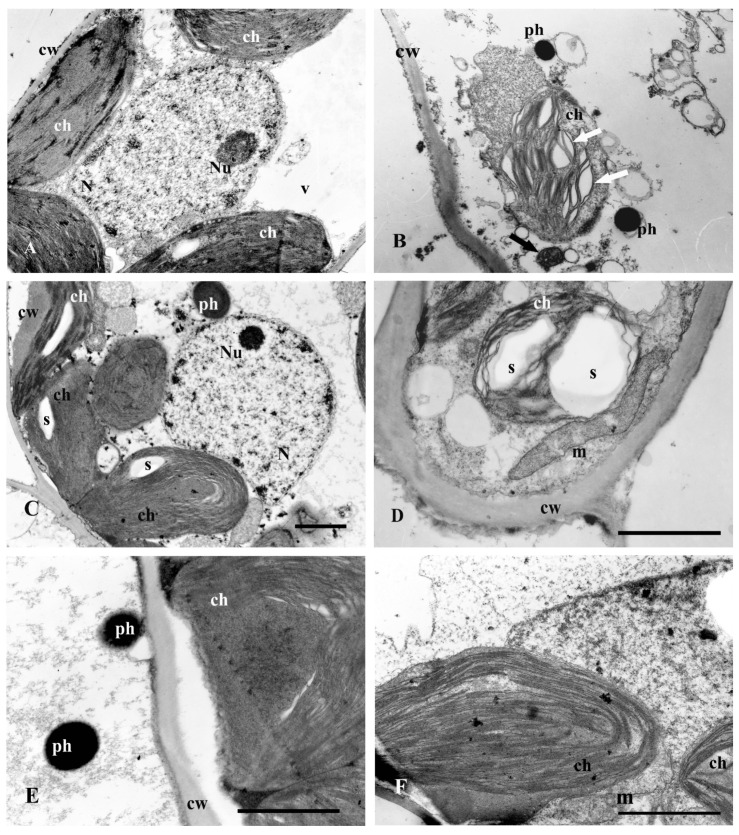
Electron micrographs of leaf spongy parenchyma cells of St. John’s wort after 7 and 17 days of culture on medium with 25 μM Cd. (**A**,**B**)—MS medium with 25 μM Cd after 7 days of culture; (**C**,**D**)—MS medium with 25 μM Cd and Se after 7 days of culture; (**E**)—MS medium with 25 μM Cd after 17 days of culture; (**F**)—MS medium with 25 μM Cd and Se after 17 days of culture. B-changed mitochondria (black arrow) and chloroplast (white arrows), description in the text. Bar = 1 μm; ch—chloroplast, cw—cell wall, m—mitochondrion, Nu-nucleolus, ph—phenolic deposits, s—starch grain, v-vacuole.

After 7 days of culture on media MS + Cd (25 µM), some degradation changes in cells, including their stronger vacuolization, the occurrence of multiple vesicles in the cytoplasm (Figure 10A) and mitochondria with swollen cristae (Figure 10A,B—black arrow) were noted. Moreover, some changes in the structure of chloroplasts were observed, like widened (Figure 10B—white arrows) or narrowed (Figure 10A) intra-thylakoidal spaces. Occasionally, some single and small starch grains were reported. After 17 days of culture, the number of phenolic compounds in cells increased (Figure 10E). Chloroplasts were often gathered in one part of the cell and had a characteristic cup shape of C. Poorly separated thylakoids as well as a few starch grains were observed.

The addition of Se to the media with Cd (25 µM) increased the number of phenolic compounds in cells, particularly in the epidermis (Figure 10C), and in enlargement of mitochondria size (Figure 10D) after 7 days of culture. After 17 days of culture, the number and size of polyphenols increased (on Cd 25 µM). The typical structure of chloroplasts has clearly discerned thylakoids and were located along the plant cell’s wall throughout the parietal cells (Figure 10F). The degradation changes observed in the media variant with Se and Cd (25 µM) were milder, both after the 7th and 17th days of culture (Figure 10C,D,F), compared to those without Se.

**Figure 11:**
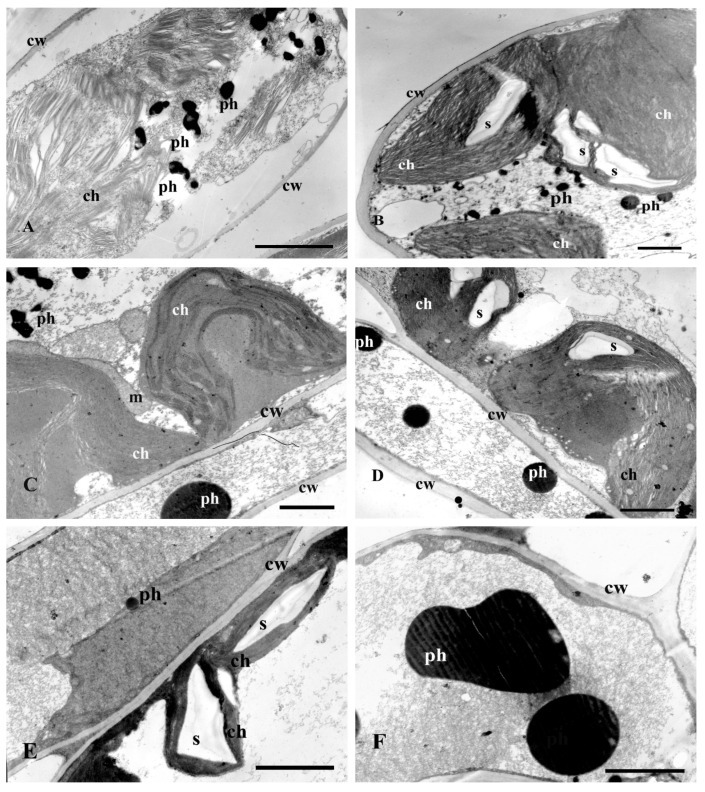
Electron micrographs of leaf spongy parenchyma cells of St. John’s wort after 7 and 17 days of culture on medium with 400 μM Cd. (**A**)—MS medium with 400 μM Cd after 7 days of culture; (**B**)—MS medium with 400 μM Cd and Se after 7 days of culture; (**C**,**D**)—MS medium with 400 μM Cd after 17 days of culture. (**E**,**F**)—plant cultured in MS medium with 400 μM Cd and Se after 17 days of culture. Description in the text. Bar = 1 μm; ch—chloroplast, cw—cell wall, m—mitochondrion, ph—phenolic deposits, s—starch grain.

Ultra-thin leaf sections of plants cultured for 7 days on MS + Cd (400 µM) media showed some structural changes in the chloroplast located in parenchyma and epidermis cells, compared to the control (MS). They were thinner, flattened, and contained no starch grains or dilatated thylakoids (Figure 11A). The parenchyma cells were often located along the plant cell’s wall and had a characteristic cup shape of C. Few beads of phenolic compounds were observed in the cytoplasm of epidermal and parenchyma cells. After 17 days of culture, the number of the starch grains in the chloroplasts as well as polyphenols in the parenchyma and epidermal cells increased (Figure 11C,D).

The addition of Se to the media resulted in a greater number of phenolic compounds, in epidermal and parenchyma cells, and of starch grains in the chloroplasts after 7 days of culture in comparison with the medium without Se (Se + Cd 400 µM) (Figure 11A,B). After 17 days of culture, both the number of starch grains (Figure 11E) and polyphenols increased, especially in the parenchyma and epidermal cells (Figure 11F). The degradation changes in chloroplasts were observed including their shape change (Figure 11E).

## 3. Discussion

There is a vast amount of data concerning the effects of Se on plants [15,16,24]. These effects depend on the Se concentration, Se form (organic, inorganic), speciation, particle size as well as plant species. To the best of our knowledge, our study is the first one on the impact of organic Se (IV) on St. John’s wort. We observed the increase in polyphenol content increased up to 2.5 fold in medium with Se, which resulted in a higher total antioxidant potential expressed as DDPH. Moreover, a 2–4 fold increase in biomass was noted. The plants had higher leaf surface and 30–50% higher leaf blade thickness compared to those grown in MS medium. Moreover, ultrastructural studies revealed the accumulation of starch granules in the chloroplasts, which may be an indicator of plant growth stimulation [26,27]. Additionally, high Se content in shoots was noted. Although higher plants do not appear to require Se, they readily take it up from their environment and incorporate it into organic compounds using sulfur-assimilating enzymes. The synthesis of selenocysteine SeCys is catalyzed by cysteine synthase and very likely takes place within the chloroplast [28]. Then, SeCys might be used in the formation of other seleno-compounds like selenomethionine, dimethylselenide (DMSe), methyl-SeCys, dimethyldiselenide (DMDSe), and elemental Se [29]. Plant species differ in their capacity to metabolize and accumulate Se, from the non-Se accumulators (<100 mg Se/kg DW), through the Se accumulators (100–1000 mg Se/kg DW) to the Se hyperaccumulators (>1000 mg Se/kg DW) [30]. We revealed St. John’s wort as the Se accumulator (550 ± 70 mg Se/kg DW), which is in accordance with Pesco’s [31] observations in shoots’ concentrations up to 398.2 mg Se/kg DW. The pathway for the assimilation of Se to SeCys in accumulators is believed to be the same as for non-accumulators. However, Se accumulators differ from non-accumulators since they metabolize the SeCys primarily into various nonprotein selenoamino acids. This metabolism probably occurs along pathways associated with the metabolism of sulfur [28].

Se is a crucial element for maintaining human health. Deficiency of Se has been correlated with higher mortality risk, cancer development, dysfunction of an immune system, and mental failure [32]. Se could be provided with food or supplemented with diet supplements. However, it should be noted that the Se content of most plants is variable and reflective of the amount available from the soil. The soil of Central European countries is deficient in selenium (Se). As a result, there are not many natural sources of Se [33]. Thus, the relatively high Se concentrations in St. John’s wort, following the exposure to organic Se, might be beneficial for consumers’ health. Recently, novel sources of selenium have been discovered such as in a selenium-enriched food like mushroom [34], rice [16], tea, or garlic [35]. Additionally, the increased polyphenol concentration can exert beneficial effects. The polyphenols are proven to have strong antioxidant properties preventing oxidative stress, which is believed to cause chronic inflammation. Chronic inflammation could mediate most chronic diseases including cancer, diabetes, cardiovascular, neurological, and pulmonary diseases [36]. Thus, culturing the plants in media with Se has both economic advantages and health benefits due to the higher plant growth rate and both increased concentration of polyphenols with strong antioxidant properties and relatively high Se level.

In our study, the mixture of seleno-triglycerides obtained by the chemical modification of sunflower oil (Selol) as a source of Se was used. No other sources of organic Se (IV) are available. The mixture revealed antimitotic and anticancerous action [37]. Although organic Se (IV) and Se (II) act differently on humans, no evidence of differences in plants exists yet. Selol reveals various effects not only on animals and humans but also fungi and plant cells [21,38,39]. One of its effects is the growth increase rate [21], which was also observed in our study.

A high concentration of heavy metals like Cd was reported to show a toxic effect on plants. In our studies, the dose-related toxic effect of Cd was observed [40]. The decline of the growth rate and degeneration in cells and organ structures was reported. Stronger vacuolization, the occurrence of multiple vesicles in the cytoplasm, as well as changes in chloroplasts and mitochondria were observed. The changes in chloroplast structure in Cd-induced stress was also observed by Filek et al. [23]. In their experiment, chloroplasts of Cd-treated plants showed an altered shape, with wavy grana and stroma thylakoids and enlarged intra-thylakoidal spaces. In addition, envelope membranes were not visible at microscopic photos of most chloroplasts. Higher plants generally produce secondary metabolites as defence-related machinery. Its enhanced production was observed as a result of abiotic stress [41]. Polyphenols constitute one of the most common molecules present in plants, with a wide range of biological activities related to their antioxidant actions [42].

Recently, the protective effect of organic Se (IV) under inorganic Se stress was observed [21]. Similarly, in our study, the addition of Se in organic form (Selol) to the medium also had a beneficial effect on St. John’s wort. The addition of Selol to the medium with Cd enhanced relative growth rates. Moreover, the degradation changes observed in media variants with Se and Cd were milder compared to those without Se. Filek et al. [23] also reported that the changes in chloroplasts originated from plants cultured in media containing the mixture of Cd + Se were not as drastic as those observed for materials prepared from plants only cultured in media containing Cd. However, some reorganization of the thylakoids and stroma was observed. The enhancement of antioxidant status following Selol treatment might explain its protective effect against abiotic stress caused by exposure, e.g., to inorganic Se or Cd [21]. In humans, Se from Selol, similarly to those from inorganic compounds, is incorporated in amino acids like SeCys [43]. After the incorporation, SeCys might be used in the biosynthesis of selenoproteins like thioredoxin reductases (TrxR), glutathione peroxidases (GPx), and thyroid hormone deiodinases (DIO). Some of them are involved in the redox regulation of intracellular signalling and redox homeostasis. In higher plants, the role of Se is different since Se is known to be a beneficial nutrient but not the essential one [44]. Plant homologues of genes encoding selenoproteins in other organisms, such as glutathione peroxidase (GPX), were shown to encode cysteine (Cys) instead of SeCys in the active site [45].

Unfortunately, Se (IV) did not decrease the Cd concentration in the plant (25 µM of Cd). Moreover, in higher concentrations of Cd (400 µM), a two-fold increase in Cd was noted. The Cd concentration can influence the metal uptake from the medium enriched with Se. For instance, inorganic Se decreased Cd content in the shoots of *Brassica Chinensis* for 10 µM of Cd, but increased for 50 µM of Cd [22]. Thus, it is possible that organic Se (IV) would influence the cadmium level at lower Cd exposure. The decrease in Cd content in shoots under Se exposure can be related to the reduction in Cd uptake or translocation towards the shoots and is observed both for inorganic and organic Se (II) [15].

## 4. Materials and Methods

### 4.1. Reagents

**Figure 12:**
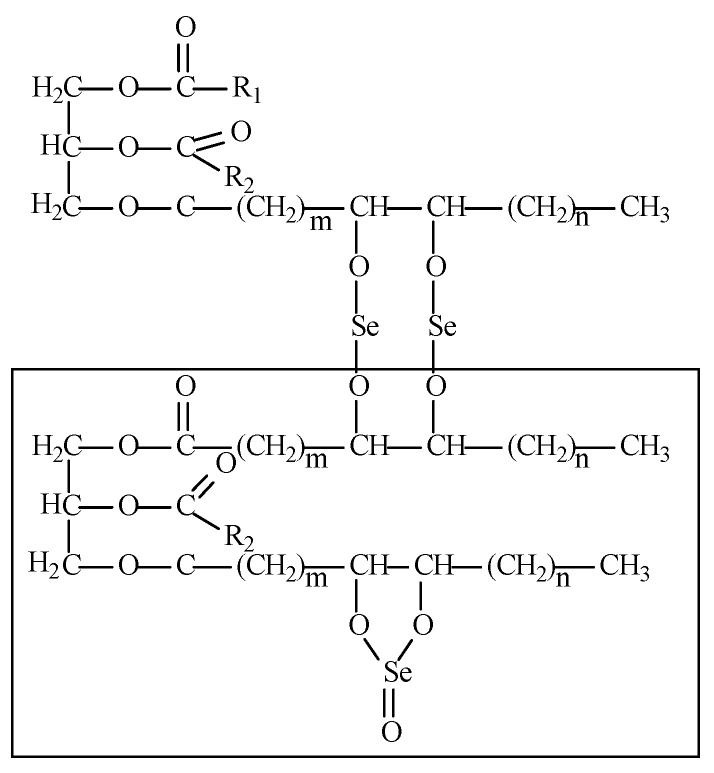
A tentative structure of Selol. The part of structure of Selol, up to 5% Se(IV), is presented in the frame (octadeca-9,11-dienoic acid 1-[7-(5-non-3-enyl-2-oxo-2λ^4^-[1,3,2] dioxaselenolan-4-yl)-heptanoyloxymethyl]-2-octadeca-9,13-dienoyloxy-ethyl ester).

All chemicals were of analytical reagent grade. The mobile phase was prepared using HPLC grade solvents (Thermo Fisher Scientific, Waltham, MA, USA) and ultrapure water (18.2 M × cm). The synthesis of Selol was carried out in the Department of Drug Analysis at the Medical University of Warsaw (Polish Patent 1999, Pl 176530). Synthesis of Selol is preceded by oxidation of a mixture of triglycerides with saturated solution of KMnO_4_ in 0.5% H_2_SO_4_. The obtained emulsion is left for several days for breaking. Hydroxyl derivatives of triglycerides are subject to esterification with selenous acid (IV) (H_2_SeO_3_) in dioxane solution. The NMR structure of Selol is presented in Figure 12. Sodium carbonate, CdCl_2_, gallic acid, and Murashige and Skoog (1962) medium (MS) were purchased in Sigma Aldrich. Ethanol (96%), Folin–Ciocalteu reagent was supplied by Avantor Performance Materials (Gliwice, Poland). Sodium hypochlorite was purchased from ACE (Bury St Edmund, UK). St. John’s wort standardized dry extract, Ph. Eur. Standardized Reference Standard (CRS), assigned value 0.050% of hypericin, was purchased from Chromadex (Los Angeles, CA, USA).

### 4.2. Plant Growth

In vitro culture was carried out in the Department of Biology and Pharmaceutical Botany, Medical University of Warsaw. *Hypericum perforatum* L. (‘Topaz’ cultivar) seeds were a kind gift from the Department of Botany, Plant Breeding and Agrotechnology Institute of Natural Fibres and Medicinal Plants in Poznań, Poland.

The seeds were placed in a tube containing detergent and a little water and shaken for about 1 min. Afterwards, the seeds were washed in running tap water and submerged in 96% ethanol for about 1 min. Then it was sterilized for 10–15 min at 5% sodium hypochlorite and shaken four times for 6–10 min in a sterile water.

Sterilized seeds used for the initiation of plantlet culture were put on MS solid medium. The medium was sterilized in an autoclave at 120 °C and 1 atm for 20 min. Seeds were sown into 100 mL conical flasks containing 30 mL of MS medium without vitamins. The seeds germinated in the dark in a culture room at 25 ± 1 °C. After six days, the flasks were moved to the culture room (T = 25 ± 1°C, 12/12 h). After 22 days of growth in MS medium without vitamins, top buds of seedlings were cut off and transferred to a 300 mL conical flask containing 50 mL of MS medium. After 36 days, shoots of plants in MS medium were transferred into 300 mL conical flasks (6 plants per flask) containing 50 mL of the following variants of MS medium: MS—the control, MS + 25μM Cd, MS + Cd 400 μM, MS + Cd 25 μM + Se, and MS + Cd 400 μM + Se. The final concentration of Se was 2 M. Cadmium was added to the medium in the form of CdCl_2_.

Shoots from three flasks were collected at 3, 7, 11, 17, and 21 days of plant growth and used for phytochemical analysis. Microscopic examination was performed using leaf fragments collected on the 7th and 17th day of the culture.

The lower concentration of Cd chosen reflects its concentration in contaminated soils (approx. 2.8 mg/kg). The highest one is much higher and not observed in the environment. However, we have chosen this concentration following Filek [23] to better observe the possible differences.

### 4.3. Phytochemical Analysis

The plants were dried in a circulating-air oven (37 ± 2 °C). The growth rate (GR) was calculated using the formula: GR = (final weight (g) − initial weight (g))/initial weight (g) × 100% [46]. Then, the plants were powdered. The target compound was extracted using methanol: water mixture (1:1, *v*/*v*) on an ultrasound bath (20 min). The extract concentration was approx. 1 mg DW/mL.

Total polyphenol content was determined by the Folin–Ciocalteu method on microplates as described previously [47]. In brief, 100 µL of water was added to the well and mixed with 25 µL of extract. Then 50 μL of 0.6 M Folin’s reagent was added to test tubes and mixed with 1 mL of 0.2 N Folin’s reagent. After 3 min, 120 μL of 1 M sodium carbonate solution was added to stop the reaction and to develop characteristic blue colour for 15 min. Absorbance was measured at 765 nm with a Synergy Mx Spectrophotometer microplate reader (Biotek, Agilent, Santa Clara, CA, USA). The total phenolic content was calculated based on the slope from serial dilution of a gallic acid standard. The final value was expressed as gallic acid equivalent.

Total antioxidant activity was determined using a 2,2-diphenyl-1-picrylhydrazyl (DPPH) assay. DPPH assay was performed by addition of 35 µL of the extract to 1250 µL DPPH (100 mg/L). After 30 min of incubation at 37 °C, the absorbance was measured at 517 nm with a Nicolet Evolution 300 (Thermo Fisher Scientific, Waltham, MA, USA). DPPH values in Trolox equivalents (TE [μmol/g]) were calculated using the standard curves, prepared in parallel with measurements, with Trolox concentration in the range 0.05–2 mM.

The total content of hypericins was determined using the HPLC method. The extract was filtered using Chromafil 0–45/25 filters. The filtrate was exposed to a halogen lamp for 10 min. HPLC was performed on a Shimadzu chromatograph consisting of a LC-10AD pump and RF10AXL fluorescence detector. Twenty microliters of the extract were introduced onto the column. Separation was performed on the Supelcosil LC-18-DB 25 cm × 4.6 mm, 5 µm column (Supelco, Sigma-Aldrich, Poznań, Polska) under isocratic conditions. The mobile phase consisted of a methanol–water mixture 50:50 (*v*/*v*). The mobile phase was pumped at a flow-rate of 1 mL/min. Chromatography was performed at 30 °C. Fluorescence excitation and emission wavelengths were set at 580 and 600 nm, respectively.

The Se and Cd concentration was determined with Avanta Ultra Z atomic spectrometer with the graphite furnace (GBC Scientific Equipment Pty, Perai, Malezja).

### 4.4. Microscopic Examination

For microscopic examination, exactly the same leaves (4th from the bottom) were used for 7- and 17-week-old plants (collected from three flasks). Small blade fragments from the middle part of the leaves were immediately fixed for 2 h in 2.5% glutaraldehyde at pH 7.2 (0.1 M cacodylic buffer) supplemented with 1% caffeine in order to improve the fixation of membranous structures according to Muller and Greenwood, as described previously [39]. Then the materials were transferred to 2% OsO4, incubated 2 h at 4 °C, dehydrated in ethanol and embedded in the mixture of epoxy resin Epon/Spurr using propylene oxide as a transition solvent [48]. Semi-thin sections (thickness of 1–2 microns) and ultra-thin (thickness of approximately 80 nm) were cut on an ultra-microtome RMC MTX Products (Boeckeler, Tucson, AZ, USA). The semi-thin sections (100 measurements for the five leaves from three different plants) were stained in 0.1% toluidine blue and examined in light microscopy (Nikon,Tokyo, Japan). The structure of the leaves was analyzed. Photographs and measurements of the thickness of the leave blades were performed as well. The ultra-thin sections (5 per leaf) were stained in saturated aqueous uranyl acetate (30 min, RT), lead citrate according to Reynolds and as described previously [49] (30 min, RT), and examined with a transmission electron microscope JEM 1400 (JEOL Co., Tokyo, Japan).

### 4.5. Statistical Analysis

The results presented are the means and standard deviations obtained for three replicates. Statistical analyses were carried out using analysis of variance (ANOVA). A *p* value less than 0.05 was considered as significant with respect to the control. Statistical tests were performed using Statistica 10 (Statsoft, Krakow, Poland). 

## 5. Conclusions

Exposure to the organic Se(IV) compound Selol did not affect hypericins but led to an increase in polyphenol content in the shoots. Growth rates remained unaffected by Cd exposure, whereas Se(IV) significantly enhanced growth rates. Additionally, adding Selol to Cd-containing medium boosted relative growth rates. Microscopic observations confirmed earlier findings of increased leaf blade thickness due to Se(IV). Furthermore, Se(IV) increased the number of starch grains in chloroplasts, whereas Cd exposure resulted in chloroplast degradation, increased cell vacuolation, and swollen mitochondrial cristae. The addition of Se(IV) mitigated these effects, even at high Cd concentration. Although Se(IV) did not alter Cd content at typical environmental levels but showed an effect at extremely high Cd concentrations.

The growth-promoting effects of Se(IV) and its accumulation in shoots of *Hypericum perforatum* L. offer significant economic and health benefits due to elevated levels of polyphenols. However, organic Se(IV) demonstrates a dual role in mitigating Cd toxicity while potentially increasing shoot accumulation in extremely high Cd environments, necessitating cautious application in heavy-metal polluted areas. Further research into the impact of organic Se(IV) on plants is justified, especially since Selol, containing Se(IV) in a triglycerides mixture, combines the organic moiety and Se(IV) effects. Despite the absence of alternative organic Se(IV) on the market, future investigations should focus on understanding its molecular and biochemical effects, ideally leveraging omics technologies such as transcriptomics, proteomics, and metabolomics. Additionally, speciation analysis is crucial for elucidating the fate of Se(IV) when administered as Selol in plants.

## Data Availability

Data available on request.
